# Is Hybrid Telehealth Model the Next Step for Private Healthcare in India?

**DOI:** 10.1177/11786329211043301

**Published:** 2021-09-01

**Authors:** Anjali Raj Westwood

**Affiliations:** York St John University, UK

**Keywords:** COVID-19, telehealth, service delivery, India

## Abstract

Irrespective of geography, the implementation of telehealth has been one of the biggest changes caused by the COVID-19 pandemic. Post the pandemic, telehealth will continue to be part of mainstream health service delivery but in a different format. This commentary investigates the hybrid model of telehealth in India. A hybrid model can help India provide accessible and affordable healthcare to a wider part of its population and support the already growing medical tourism industry. The challenges to this revolve around digital education for patients and providers, integration of technology into existing care pathways, infrastructural investment and in creating seamless systems. It is a scalable and profitable model that must be seriously considered in the post pandemic world.

## Introduction

As per World Health Organisation,^1^ telehealth is the ‘delivery of health care services, where patients and providers are separated by distance. Telehealth uses ICT for the exchange of information for the diagnosis and treatment of diseases and injuries, research and evaluation, and for the continuing education of health professionals’. Telemedicine or telehealth can be classified based on mode of communication as (i) audio, video or text-based; (ii) timing of information transmitted as synchronous (real time) or asynchronous (store and forward) exchange; (iii) purpose of consult as first time or follow up and (iv) nature of the relationship of parties on the call such as: patient and medical practitioner, caregiver and medical practitioner, medical practitioner and medical practitioner or health worker and medical practitioner.^2^ It can also be classified based on the type of health services to be delivered (mental health, dermatology, general medicine, physiotherapy, etc.).

Telehealth aims to ensure equitable services to everyone, is cost-effective, provides safety to both patient and doctors during pandemics, and offers timely and faster care.^3^ Though there are years of research on effectiveness, efficiency, practicality and feasibility of telehealth, COVID-19 has put the practice of telehealth in the limelight. Hospitals and clinics had to change their operating model in a swift manner to accommodate patients needing health services. Global online doctor consultation market has been predicted to be worth $16 billion by 2026 with a Compound Annual Growth Rate (CAGR) of 26.6%.^4^

## Telehealth in India

The Indian telemedicine market is projected to reach $5.5 billion by 2025^5^ and the pandemic has further fuelled the growth of this market. In March (2020), the Ministry of Health and Family Welfare (MoHFW) – in collaboration with NITI Aayog and Board of Governors (BoG), and the Medical Council of India (MCI) instituted a regulatory framework for telemedicine in India.^2^ The framework provides detailed guidelines and recommendations to care providers on digital care pathways, prescriptions and more.

India has a high disease burden of cardiovascular, diabetes and respiratory disease, killing 4 million Indians aged 30 to 70 every year (as on 2016).^6^ The onset occurs around 45 years of age as compared to 55 in developed countries. High blood pressure, high total cholesterol, high fasting plasma glucose and high body-mass index contributes to co-morbidities. All the above non communicable diseases (NCD) lend well to telemedicine as patients can be trained to self-report blood pressure/sugar/weight during online consultations (initial and follow ups).

Increase in mental health issues due to the COVID-19 pandemic has been observed across the world. An Indian online survey conducted in 2020 reported that out of 396 participants, 40.5% participants reported anxiety/depressive symptoms, 74.1% reported moderate stress and 71.7% reported poor wellbeing.^7^ This increase in need for mental health support has been picked up by the telemedicine providers who offer varying levels of counselling and therapy.

In August 2020, the Union Health Ministry of India announced that in less than 9 months since its inception, over 100 000 free online consultations had been conducted on the government platform called eSanjeevani.^8^ An interesting trend in India was observed amongst the over 50’s age group which saw an exponential rise of 502% during this past year.^9^ This increase has been attributed to acceptance of new technology during the pandemic as a necessity to navigate health services, and the favourable option of round the clock care when in prolonged isolation. As a high-risk population in relation to the COVID-19 pandemic, another key factor can be the relative risk of physical appointments versus digital appointments.

Global consulting firm McKinsey^10^ reported that India can save between $4 and $5 billion in healthcare costs with widespread implementation of telemedicine. There are a range of players in the Indian telemedicine market. Some are independent platforms (eg, practo, doc online, credihealth, indiaopd, etc.) and some are linked to a hospital (eg, apollo health, cloudnine, fortis healthcare).

There is no question about the growth potential for this industry. However, pure telehealth cannot entirely address health service delivery. Telehealth is suitable for initial consults, triage and follow up. However, physical interaction between the care providers and the patient, is key to diagnosis and treatments. For example, a GP can digitally speak to a patient complaining of stomach pain for initial assessment, only if deemed necessary, the patient would need to come in for a test/scan. Depending on the results, the follow up can be digital or in-person. An integrated pathway of virtual and in-person care will be necessary for long-term efficient care delivery. In this commentary, I will look at hybrid telehealth models, its benefits and challenges, taking into consideration the Indian context.

## What is a Hybrid Telehealth Model?

Furuya et al^11^ describes the hybrid model as a service that consists of alternating in- person and telehealth visits. New patient appointments are virtual, and initial follow up appointments are in-person. New patient appointments often place heavy emphasis on detailed history. This can be completed via telehealth which, by design, is more conversation focussed. Follow-up appointments are initially scheduled as in-person visits, given that many of these appointments require problem-focussed physical exams and diagnostic work. Subsequent visits can be telehealth if this seems appropriate to the patient and the doctor.

## Advantages of a Hybrid Model

The hybrid model has many advantages. It allows for triage and screening to prevent unnecessary contact, for observation of the patient in their home environment, saves travel time and cost, and increases affordable accessibility to specialists. As mentioned previously, hybrid models can create a continuum of care that pure telehealth cannot. Screening and diagnostic tests, physical examinations and treatments do not fit in with a pure telehealth model, but it does work in a hybrid model. Hybrid model can create efficient care pathways.

Telehealth can be home based, or clinic based and hybrid models utilising clinic based telehealth can break geographical boundaries in India. For example, the eSanjeevani model as reported by Kaul,^8^ operates on a hub and spoke model in the rural areas, with medical colleges as hubs and primary health centres as spokes. This allows for patients in rural areas to access specialist advice via telehealth from medical colleges without travel and time cost. Post basic consultations and primary level diagnostics, the doctors can determine whether the patient needs to travel large distances to medical colleges for speciality treatment. In private healthcare, cost of all in-person visits is expensive. This can lead to missed visits and usage of health services. The hybrid model will be more affordable as tele consults can be charged at a fraction of the usual cost. This, I believe, will attract more patients; patients will keep appointments and they will incur a lower travel and time cost overall.

The most profitable part of telehealth is the international segment that opened during the pandemic. For example, Apollo hospitals offer online consultations priced from USD 55 for all medical specialities.^12^ This model is highly profitable for the hospitals as local consultations at the same hospital would cost a patient between USD 7 and USD 15.

Pre-Covid, India was gaining its popularity for medical tourism. Medical tourism is defined as ‘a practice of travelling to countries offering competitive advantage of world class treatment at low cost regarding wellness and health related treatment’.^13^ The main objective of medical tourists are long term healthcare treatments or elective surgeries. These can be further split into diagnostic, clinical, invasive or lifestyle conditions.^14^ As per Bagga et al,^15^ 427 000 patients medical tourists visited India in 2017 and it generated $3 billion USD. Assuming that this trend will rise again in the next 5 years, post Covid, hospitals can achieve greater patient experience and seamless transition of care by utilising a hybrid telehealth model where initial consults and doctor-patient interaction can happen weeks before they arrive in India for their treatment.

## Challenges to Integration and Implementation of a Hybrid Model

As with any new model, there are always nuances that evolve during implementation. Disadvantages to this model include limitations on incorporating education during the telehealth visit and variance in technological literacy and access of our patient population.

India has a wide range of socio-economic levels which create a gap in accessibility to technology and digital literacy.^16^ A risk that can’t be left out is of miscommunication of symptoms, prognosis, or prescriptions amongst the patients with digital literacy challenges. There can be detrimental effects if telehealth is being used as the first point of contact between patients and clinicians, and there is a struggle to establish a strong patient-doctor relationship.

Interpretations of prescriptions should be given high importance as any error by the patients, or the chemists can lead to disastrous results.^3^ One way to partially mitigate this risk is by addressing this step of the treatment process during one of the in-person visits. A second option is to discourage providing prescriptions via conversation and instead opt for fixed format email prescriptions.^3^ The current regulatory guidelines in India^2^ segregates drugs based on risk level. The low-risk drugs can be prescribed without an in-person consult. The prescriptions can be a photo, scan or a digital copy sent via email or any messaging platform. The process can be simplified for the patient if the care providers have a direct link to remote prescribe to a pharmacy in the patient’s catchment area, and all the patient or care giver needs to do is collect it. This model is approved by the regulatory guidelines but there is no data on the range of adoption of this method by clinical practitioners.

The number of smartphone users in India was estimated to reach over 760 million in 2021 and is projected to reach over 973 million by 2025.^17^ However, the quality of internet connections across the country are not stable enough.^18^ Metropolitan cities and towns will have better internet access than other areas, which will lead to usage of telehealth systems predominantly amongst the urban population. Until internet connections don’t stabilise across rural areas and smaller towns, the hybrid telehealth system will not truly address the issue of geographical accessibility to specialist healthcare. The eSanjeevani hub and spoke model connecting rural areas to specialist medical colleges discussed in the previous section can help address this challenge. Further infrastructural investment is needed to set up tech-enabled clinics in areas where individual smartphone penetration, networks or digital literacy is low.

There are a range of innovative start-ups and organisations providing seamless software for telemedicine. Platforms like practo or credihealth have easy to use mobile or web applications that can be downloaded and used. As India’s healthcare system is a mix of private and public, there is a lack of universal electronic health records (EHR). Each hospital has their own records which affect transfer of data if a patient chooses to switch hospitals or clinics. A benefit of platform-based telemedicine is the option to store and share data with a range of other doctors who may or may not be from the same hospital or geographical location. In a hybrid healthcare format, the hospitals will have an easier transition as records can be accessed on their website or in the hospital. How this will work for platform-based care providers is yet to be seen.

Healthcare workers have historically not been trained in digital skills.^18^ Training programmes will have to be set up to upskill the existing workforce towards efficient and accurate usage of telehealth software. Along with digital training to use telemedicine platforms and softwares, healthcare workers would need training in establishing patient rapport via a virtual medium. Six Indian teaching hospitals used technology for surgical training in the field of endocrinology, gastrointestinal surgery and urology.^19^ Yadav also reports that minimal numbers of tele-mentoring guided surgeries have been conducted. Though increasingly common in the western countries, use of technology for surgical training and practice is currently low in India, but is expected to grow in the future.

## Conclusion

Along with the rest of the world, India is looking at a shift in health service practices. The hybrid model isn’t entirely new. Pre-pandemic, the challenges and fears regarding the hybrid model were on the telehealth side of the spectrum. There was a lack of trust on digital technology to efficiently support care providers to deliver a service which has been historically in-person led to resistance. COVID-19 has made telehealth irreplaceable, and ready to be accepted into mainstream service delivery by the healthcare providers. India’s high NCD burden can be supported efficiently with hybrid models.

Due to the recency of the topic, a limitation of this paper is that organisational reports, white papers and web based data has been used alongside journal articles. Three areas need further research: The acceptance and readiness of doctors and patients to shift to hybrid models post the COVID-19 pandemic, the infrastructural readiness to adopt hybrid healthcare and the skill gaps among the healthcare staff who will potentially operate within the model.

This new model can help in efficient and effective health service delivery in a country like India with its vast geography leading to healthcare accessibility challenges. Integrating digital skills within healthcare education, creating affordable private healthcare through digital innovation, regular amendments to regulatory processes, developing sustainable infrastructure, implementing systemic changes and establishing better connections between ministries of health and other parties such as ministries of communication and others to facilitate distribution of hardware and software, in addition to improving internet connection speed, will be the key to transforming Indian healthcare.
